# Association of *PALB2* sequence variants with the risk of familial and early-onset breast cancer in a South-American population

**DOI:** 10.1186/s12885-015-1033-3

**Published:** 2015-01-31

**Authors:** Yessica Leyton, Patricio Gonzalez-Hormazabal, Rafael Blanco, Teresa Bravo, Ricardo Fernandez-Ramires, Sebastian Morales, Natalia Landeros, Jose M Reyes, Octavio Peralta, Julio C Tapia, Fernando Gomez, Enrique Waugh, Gladys Ibañez, Janara Pakomio, Gilberto Grau, Lilian Jara

**Affiliations:** 1 Human Genetics Program, Institute of Biomedical Sciences (ICBM), School of Medicine, University of Chile, Av. Independencia 1027, Santiago, Chile; 2 National Cancer Society (Corporación Nacional del Cáncer –CONAC-), Santiago, Chile; 3 Research Institute in Dental Sciences, School of Odontology, University of Chile, Sergio Livingstone Pohlhammer 943, Santiago, Chile; 4 Clínca Las Condes, Santiago, Chile; 5 Department of Gyneacology and Obstetrics, School of Medicine, University of Chile, Av Santa Rosa 1234, Santiago, Chile; 6 Cell Transformation Laboratory, Institute of Biomedical Sciences (ICBM), School of Medicine, Unversity of Chile, Av. Independencia 1027, Santiago, Chile; 7 Clínica Santa María, Santiago, Chile; 8 Clínica Dávila, Av. Recoleta 464, Santiago, Chile; 9 Hospital San José, San José 1196, Santiago, Chile

**Keywords:** Familial breast cancer, PALB2, BRCA1/2-negative families, South-American population

## Abstract

**Background:**

Germline mutations in *PALB2* have been identified in approximately 1% of familial breast cancer (BC) in several populations. Nevertheless its contribution in the South-American population is unknown. The goal of this study was to determine the prevalence of *PALB2* mutations in the Chilean population.

**Methods:**

100 Chilean *BRCA1/2*-negatives familial BC cases were included for the *PALB2* mutation analysis. We use conformational sensitive gel electrophoresis and direct sequencing. Using a case-control design, we studied the identified variants in 436 BC cases and 809 controls to evaluate their possible association with BC risk.

**Results:**

No pathogenic mutations were detected. We identified three variants, the variant c.1861C > A not previously described was found in one of the 436 cases and none of the 809 controls. The bioinformatic analyses indicate that this variant probably is not pathogenic. *PALB2* c.1676A > G (rs152451A/G) and c.2993C > T (rs45551636C/T) variants were significantly associated with increased BC risk only in cases with a strong family history of BC (OR = 1.9 [CI 95% 1.3-2.8] p < 0.01 and OR = 3.3 [CI 95% 1.4-7.3] p < 0.01, respectively). The rs152451A/G-rs45551636C/T composite genotype produce increase of the BC risk in cases with a strong family history of BC (OR = 3.6 [CI 95% 1.7-8.0] p = 0.003). The rs152451-G/rs45551636-C and rs152451-G/rs45551636-T haplotypes were associated with an increased BC risk only in cases with a strong family history of BC (OR = 1.6 [CI 95% 1.0-2.5] p = 0.05 and OR = 3.7 [CI 95% 1.8-7.5] p < 0.001, respectively).

**Conclusion:**

Our results suggest that *PALB2* c.1676A > G and c.2993C > T play roles in BC risk in women with a strong family history of BC.

**Electronic supplementary material:**

The online version of this article (doi:10.1186/s12885-015-1033-3) contains supplementary material, which is available to authorized users.

## Background

Genetic factors play an important role in breast cancer (BC) development, including the presence of *BRCA1*, *BRCA2*, *ATM,* and other genes [1], but only 5% of BC incidence can be explained by mutation in these high-penetrance genes [2]. Moreover, these genes are responsible for only about 16-20% of the risk for familial BC. Therefore, the genetic basis of 80% of familial cases remains unexplained [3]. The remaining risk is likely to involve mutations in moderate- and/or low-penetrance susceptibility genes.

The PALB2 (partner and localizer of BRCA2) protein interacts with BRCA2, stabilizing the intranuclear accumulation of BRCA2 proteins at sites of DNA damage [4]. PALB2 is also recruited by BRCA1 in response to DNA damage and serves as a linker between BRCA1 and BRCA2, necessary for *BRCA2*-mediated homologous-recombination repair [5,6]. Thus, *BRCA1*, *BRCA2*, and *PALB2* are key BC susceptibility genes that function together in the same DNA damage response pathway [5,7]. Biallelic loss of *PALB2* causes increased predisposition to cancers, increased sensitivity to DNA-damaging agents, and Fanconi anemia [8].

Germline *PALB2* mutations are rare, but have been associated with increased risk for breast and other cancers. In families with multiple breast cancer cases, germline *PALB2* mutations have been reported in populations from Western Europe, the United Kingdom, Finland, French Canada, and Australia [9-12]. In familial BC, germline *PALB2* mutations are associated with a 2.3- to 6.0-fold increased risk for BC [9,13,14]. Germline *PALB2* mutations have also [13] been reported in a lower frequency in unselected female BC cases from Finland, French Canada, Australia, China, Malaysia, and Singapore [10-12,15,16]. No *PALB2* mutations have been identified in Jewish families of either Ashkenazi or non-Ashkenazi origin [17,18]. The contribution of *PALB2* mutations to BC in the South American population is unknown. The Chilean population is the result of admixture between Asian and Spanish populations; therefore, whether germline *PALB2* mutations contribute to Chilean BC risk is unknown.

In this study, we screened the coding regions and exon-intron boundaries of the *PALB2* gene in *BRCA1/2*-negative women with familial BC, to determine the contribution of *PALB2* variation in the Chilean population. Also, using a case-control design, we studied the c.1676A > G (exon 4), c.1861C > A (exon 5), and c.2993C > T (exon 9) variants to evaluate their possible association with BC susceptibility.

## Methods

### Families

A total of 436 BC patients belonging to 436 high-risk *BRCA1/2*-negative Chilean families were selected from the files of the Servicio de Salud del Area Metropolitana de Santiago, Corporación Nacional del Cáncer (CONAC) and other private services in the Metropolitan Area of Santiago. All index cases were tested for *BRCA1* and *BRCA2* mutations as described [19]. Pedigrees were constructed on the basis of an index case considered to have the highest probability of being a deleterious mutation carrier. None of the families met the strict criteria for other known syndromes involving BC, such as Li-Fraumeni, ataxia-telangiectasia, or Cowden disease.

Table 1 shows the specific characteristics of the families selected according to the inclusion criteria. All families participating in the study self-reported Chilean ancestry dating from several generations, after extensive interviews with several members of each family from different generations. In the selected families, 12.4% (54/436) had cases of bilateral BC; 8.0% (35/436) had cases of both BC and ovarian cancer (OC); and 2.1% (9/436) had male BC. In the BC group, the mean age of diagnosis was 42.7 years, and 75.2% had age of onset < 50 years.

**Table 1:** **Inclusion criteria for the families included in this study**

Inclusion criteria	Families n (%)
Three or more family members with breast and/or ovarian cancer	124 (28.4%)
Two family members with breast and/or ovarian cancer	149 (34.2%)
Single affected individual with breast cancer ≤ age 35	84 (19.3%)
Single affected individual with breast cancer between age 36 and 50	79 (18.1%)
TOTAL	436 (100%)

This study was approved by the Institutional Review Board of the School of Medicine of the University of Chile. Informed consent was obtained from all participants.

### Control population

The sample of healthy Chilean controls (n = 809) was recruited from CONAC files. DNA samples were taken from unrelated individuals with no personal or familial history of cancer who gave their consent for anonymous testing. These individuals were interviewed and informed as to the aims of the study. DNA samples were obtained according to all ethical and legal requirements. The control sample was matched by age and socioeconomic strata with respect to the cases. Over 90% of cases and controls lived in the city of Santiago.

### Mutation analysis

Genomic DNA was extracted from peripheral blood lymphocytes of 436 cases belonging to the high-risk selected families and 809 controls. Samples were obtained according to the method described by Chomczynski and Sacchi [20].

### *PALB2* complete sequence analysis

This analysis was performed in 100 cases belonging to families with a) three or more family members with breast and/or ovarian cancer and b) two family members with breast and/or ovarian cancer. The whole coding sequence and exon–intron boundaries of the *PALB2* gene were amplified by polymerase chain reaction (PCR) using previously-described primers [21]. For exon 9, we designed primers using Web Primer3 version 0.4.0 [22]. We amplified 16 amplicons, whose sizes are shown in Additional file 1: Table S1. The fragments obtained were analyzed for sequence variants using conformational sensitive gel electrophoresis (CSGE) [23]. Amplified samples were denaturated at 95°C for 5 minutes and 65°C for 30 minutes to generate heteroduplex. The products were diluted 1:2 in sucrose buffer and loaded in a partially denaturing MDE® gel (Cambrex, UK) at constant power of 7 W during different time periods depending on the size of the fragment. Gels were silver-stained and dried on a vacuum gel dryer.

Any fragment showing a mobility shift was directly sequenced, to identify the variant. Sequencing was performed in an ABI 3730XL automated fluorescence-based cycle sequencer and a BigDye v.3.1 terminator system (Applied Biosystems, Foster City, CA). The sequences utilized for naming PALB2 sequence variants, obtained from the NCBI RefSeq database, were NM_024675.3 (transcript) and NP_078951.2 (protein).

### *PALB2* rs152451 (1676A > G), rs45551636 (2993C > T), and c. 1861C > A analysis

Genotyping for rs152451 (c.1679A > G), rs45551636 (c.2993C > T), and c.1861C > A was carried out using TaqMan SNP Genotyping Assays (Applied Biosystems, Foster City, CA) (assay ID C_2392113_10, C_86371270_10, and a customized assay, respectively). The reaction was performed in a 10 uL final volume containing 5 ng of genomic DNA, 1X TaqMan Genotyping Master Mix, and 1X TaqMan SNP Genotyping Assay. Polymerase chain reaction was carried out in a StepOnePlus Real-Time PCR System (Applied Biosystems, Foster City, CA). The thermal cycles were initiated for 10 minutes at 95°C, followed by 40 cycles each of 92°C for 15 seconds and 60°C for 1 minute. Each genotyping run contained DNA controls confirmed by sequencing. The alleles were assigned using the SDS 2.2 software (Applied Biosystems, Foster City, CA). As a quality control, we repeated the genotyping on ~10% of the samples, and all genotype scoring was performed and checked separately by two reviewers unaware of the case-control status.

### Bioinformatics and statistical analyses

The Hardy-Weinberg equilibrium assumption was assessed in the control sample using a goodness-of-fit chi-square test (HWChisq function included on “HardyWeinberg”.package v.1.4.1). Fisher’s exact test was used to test the association of genotypes and/or alleles between cases and controls. *p* < 0.05 was used as the criterion of significance. The odds ratios (OR) and their 95% confidence interval (CI) were calculated to estimate the strength of the association between cases and controls (odds ratio fisher function included on “epitools” package v.0.5-6). Haplotype estimation was carried out using UNPHASED v.3.1.5 software, which uses a maximum likelihood approach [24]. *In silico* analyses of the effect of missense mutations on protein function were carried out using PolyPhen-2 (Polymorphism Phenotyping version 2) [25], SIFT [26] and PROVEAN [27]. To evaluate protein stability we used I-Mutant 3.0 tools (http://gpcr2.biocomp.unibo.it/cgi/predictors/I-Mutant3.0/I-Mutant3.0.cgi). We obtained the wildtype structure of the *PALB2* protein from RASMOL (http://rasmol.org/) and used MODELLER v.9.12 (http://salilab.org/modeller/) to analyze the 3D-structure and function of the *PALB2* protein.

## Results

We analyzed the complete coding sequence and splice-boundary region of *PALB2* in 100 probands from BC families negative for *BRCA1* and *BRCA2* point mutations with the aim of identifying *PALB2* sequence variation in a Chilean population. We identified three sequence variants: c.1676A > G (p.Q559R, rs152451) (exon 4), c.1861C > A (p.P621M) (exon 5), and c.2993C > T (p.G998E, rs45551636) (exon 9). The variants detected were analyzed in 436 BC *BRCA1/2*-negative cases and 809 controls. For the analysis, the whole sample was subdivided into two groups: cases belonging to families with two or more family members with BC and/or OC (n = 273) (subgroup A) and non-familial early-onset BC (≤50 years) (n = 163) (subgroup B).

The variant c. 1861C > A, not previously described in the literature nor in the *PALB2* variation database (http://www.lovd.nl/PALB2), was found in only one of the 436 cases and none of the 809 controls. Therefore, this mutation corresponds to a new mutation not previously described.

The c.1676A > G and c.2993C > T variants correspond to previously-described variants (rs152451 and rs45551636, respectively). Table 2 shows the genotype distributions and allele frequencies of rs152451 and rs45551636 variants in the whole data set and in subgroups A and B with respect to the controls. The observed genotype frequencies for the two variants were all in Hardy–Weinberg equilibrium in the controls (P = 0.46 for rs152451, P = 0.68 for rs45551636, respectively).

**Table 2:** **Genotype and allelic frequencies of PALB2 rs152451 and rs45551636 in** ***BRCA1/2*** **-negative breast cancer cases and controls**

		All BC cases (n = 436)			Families with ≥ 2 BC and/or OC cases (n = 273)			Single affected, diagnosis ≤ 50 years (n = 163)		
Genotype or allele	Controls (%) (n = 809)	BC cases (%)	*P*value^a^	OR [95% CI]	BC cases (%)	*P*value^a^	OR [95% CI]	BC cases (%)	*P*value^a^	OR [95% CI]
rs152451										
AA	674 (83.3%)	351 (80.5%)	-	1.0 (ref)	210 (76.9%)	0	1.0 (ref)	141 (86.5%)	-	1.0 (ref)
AG	127 (15.7%)	79 (18.1%)	0.26	1.2 [0.9-1.6]	60 (22.0%)	**0.02**	**1.5 [1.1-2.2]**	19 (11.7%)	0.23	0.7 [0.4-1.2]
GG	8 (1.0%)	6 (1.4%)	0.57	1.4 [0.4-4.8]	3 (1.1%)	0.73	1.2 [0.2-5.1]	3 (1.8%)	0.41	1.8 [0.3-7.6]
AG + GG	135 (16.7%)	85 (19.5%)	0.21	1.2 [0.9-1.6]	63 (23.1%)	**0.02**	**1.5 [1.1-2.1]**	22 (13.5%)	0.35	0.8 [0.5-1.3]
A	1475 (0.91)	781 (0.90)	-	1.0 (ref)	480 (0.88)	0	1.0 (ref)	301 (0.92)	-	1.0 (ref)
G	143 (0.09)	91 (0.10)	0.19	1.2 [0.9-1.6]	66 (0.12)	**0.03**	**1.4 [1.0-1.9]**	25 (0.08)	0.59	0.9 [0.5-1.3]
rs45551636										
CC	786 (97.2%)	413 (94.7%)	-	1.0 (ref)	255 (93.4%)	-	1.0 (ref)	158 (96.9%)	-	1.0 (ref)
CT	23 (2.8%)	22 (5.0%)	0.06	1.8 [1.0-3.5]	17 (6.2%)	**0.01**	**2.3 [1.1-4.5]**	5 (3.1%)	0.80	1.1 [0.3-2.3]
TT	0 (0.0%)	1 (0.2%)	-	-	1 (0.4%)	-	0	0 (0.0%)	-	-
CT + TT	23 (2.8%)	23 (5.2%)	**0.04**	**1.9 [1.0-3.6]**	18 (6.6%)	**0.01**	**2.4 [1.2-4.7]**	5 (3.1%)	0.80	1.1 [0.3-2.3]
C	1595 (0.99)	848 (0.97)	-	1.0 (ref)	527 (0.97)	-	1.0 (ref)	321 (0.98)	-	1.0 (ref)
T	23 (0.01)	24 (0.03)	**0.03**	**2.0 [1.1-3.7]**	19 (0.03)	**0.01**	**2.5 [1.3-4.8]**	5 (0.02)	0.80	1.1 [0.3-2.9]

*BC* breast cancer, *OC* ovarian cancer, *OR* odds ratio, *CI* confidence interval.

^a^Fisher’s exact test.

Bold values are statistically significant (*P* < 0.05).

In the single locus analysis, the genotype and allele distribution for rs152451 did not indicate an association of this variant with increased BC risk either in the whole sample or in subgroup B. However, in the familial BC cases (subgroup A), the minor allele frequency (MAF) (allele G) was higher in cases than in controls (0.12 and 0.09, respectively, p = 0.03). Furthermore, in subgroup A, allele G carriers (AG + GG) were associated with a significantly increased BC risk (OR = 1.5 [CI 95% 1.1-2.1], p = 0.02) (Table 2). We also analyzed the relationship between rs152451 and BC risk within patients with BC familial history according the number of BC cases in the family (Table 3). No association between rs152451 and BC risk was found in cases belonging to families with two BC and/or OC cases. Nevertheless, BC risk was significantly increased in cases belonging to families with 3 or more members affected by BC and/or OC. In these families, the frequency of allele G was 0.16 in BC cases versus 0.09 in controls (OR = 1.9 [CI 95% 1.3-2.8] p < 0.01), and G allele carriers (AG and AG + GG) were associated with a significantly increased BC risk (OR = 2.0 [CI 95% 1.3-3.2] p < 0.01).

**Table 3:** **Genotype and allelic frequencies of PALB2 rs152451 and rs45551636 according the number of BC cases in the families in** ***BRCA1/2*** **-negative breast cancer cases and controls**

		Families with 2 BC and/or OC cases (n = 124)			Families with ≥ 3 BC and/or OC cases (n = 149)		
Genotype or allele	Controls (%) (n = 809)	BC cases (%)	*P*value^a^	OR [95% CI]	BC cases (%)	*P*value^a^	OR [95% CI]
rs152451							
AA	674 (83.3%)	88 (71.0%)	-	1.0 (ref)	122 (81.9%)	-	1.0 (ref)
AG	127 (15.7%)	33 (26.6%)	0.47	1.2 [0.7-1.9]	27 (18.1%)	**<0.01**	**2.0 [1.2-3.1]**
GG	8 (1.0%)	3 (2.4%)	-	-	0 (0.0%)	0.13	2.9 [0.5-12.2]
AG + GG	135 (16.7%)	36 (29.0%)	0.63	1.1 [0.7-1.8]	27 (18.1%)	**<0.01**	**2.0 [1.3-3.2]**
A	1475 (0.91)	209 (0.84)	-	1.0 (ref)	271 (0.91)	-	1.0 (ref)
G	143 (0.09)	39 (0.16)	0.91	1.0 [0.6-1.6]	27 (0.09)	**<0.01**	**1.9 [1.3-2.8]**
rs45551636							
CC	786 (97.2%)	113 (91.1%)	-	1.0 (ref)	142 (95.3%)	-	1.0 (ref)
CT	23 (2.8%)	10 (8.1%)	0.30	1.7 [0.6-4.1]	7 (4.7%)	**<0.01**	**3.0 [1.2-6.8]**
TT	0 (0.0%)	1 (0.8%)	-	-	0 (0.0%)	-	-
CT + TT	23 (2.8%)	11 (8.9%)	0.30	1.7 [0.6-4.1]	7 (4.7%)	**<0.01**	**3.3 [1.4-7.3]**
C	1595 (0.99)	236 (0.95)	-	1.0 (ref)	291 (0.98)	0	1.0 (ref)
T	23 (0.01)	12 (0.05)	0.30	1.7 [0.6-4.1]	7 (0.02)	**<0.01**	**3.5 [1.6-7.5]**

*BC* breast cancer, *OC* ovarian cancer, *OR* odds ratio, *CI* confidence interval.

^a^Fisher’s exact test.

Bold values are statistically significant (*P* < 0.05).

With respect to rs45551636, the genotype and allele distribution did not indicate an association of this variant with non-familial early-onset BC (≤50 years) (Table 2). Nevertheless, in the whole sample, the MAF (T allele) was higher in cases (0.03) than controls (0.01), and the difference was statically significant (OR = 2.0 [CI 95% 1.1-3.7], p = 0.03). This result indicates that the T allele is associated with a significantly increased BC risk. We also observed increased risk of BC for T allele carriers (CT + TT) in the whole sample and in subgroup A (OR = 1.9 [CI 95% 1.0-3.6] p = 0.04 and OR = 2.4 [CI 95% 1.2-4.7] p = 0.01, respectively) (Table 2). When we analyze the effect of allele T considering the number of BC cases in the families, no association between rs45551636 and BC risk was found in cases belonging to families with two BC cases. Nevertheless, BC risk increased 3.0-fold in the heterozygous group (OR = 3.0 [CI 95% 1.2-6.8] p < 0.01) and 3.3-fold in allele T carriers (CT + TT) belonging to families with 3 or more cases of BC (OR = 3.3 [CI 95% 1.4-7.3] p < 0.01) (Table 3).

Table 4 shows the composite genotype analysis for rs152451 and rs45551636. The rs152451 A/G - rs45551636 C/T composite genotype showed a higher frequency in cases compared to controls in the whole sample and in subgroup A (OR = 1.9 [CI 95% 1.0–3.6] p = 0.04 and OR = 2.6 [CI 95% 1.4–4.5] p = 0.01 respectively) (Table 4). Nevertheless, the frequency of A/G-C/T composite genotype did not differ between non-familial early-onset BC (≤50 years) versus controls (OR = 0.9 [CI 95% 0.3–2.7] p = 1.0). When we analyzed the relationship between the composite genotype and BC risk within patients with familial BC history according the number of BC cases in the family (Table 4), we observed that the A/G-C/T composite genotype, which includes the two risk alleles, was associated with a significantly increased BC risk both in cases belonging to families with two or more BC and/or OC cases and in cases belonging to families with three or more BC and/or OC cases, with a stronger effect of the composite genotype in the latter group of cases "(OR = 2.6 [CI 95% 1.4-5.0] p = 0.01) and OR = 3.6 [CI 95% 1.7-8.0] p = 0.003, respectively). No association was observed in the families belonging to subgroup B. In conclusion, the A/G-C/T composite genotype produces a higher increase of BC risk in cases with a strong family history of BC and/or OC. This finding raises the possibility that these variants are in strong linkage disequilibrium.

**Table 4:** **Composite genotype frequencies for rs152451 and rs45551636 PALB2 variants in** ***BRCA1/2*** **negative breast cancer cases and controls**

		All BC cases (n = 436)			Families with ≥ 2 BC and/or OC cases (n = 273)			Families with ≥ 3 BC and/or OC cases (n = 124)			Single affected, diagnosis ≤ 50 years (n = 163)		
Composite genotype (rs152451-rs45551636)	Controls (%) (n = 809)	BC cases (%)	*P*value^a^	OR [95% CI]	BC cases (%)	*P*value^a^	OR [95% CI]	BC cases (%)	*P*value^a^	OR [95% CI]	BC cases (%)	*P*value^a^	OR [95% CI]
A/A-C/C	674 (83.3%)	351 (80.5%)	-	1.0 (ref)	210 (76.9%)	-	1.0 (ref)	88 (71.0%)	0	1.0 (ref)	141 (86.5%)	-	1.0 (ref)
A/G-C/C	106 (13.1%)	58 (13.3%)	0.79	1.1 (0.7-1.5)	43 (15.8%)	0.18	1.3 (0.9-1.9)	23 (18.6%)	**0.06**	**1.7 (1.0-2.7)**	15 (9.2%)	0.19	0.7 (0.4-1.2)
A/G-C/T	21 (2.6%)	21 (4.8%)	**0.04**	**1.9 (1.0-3.6)**	17 (6.2%)	**0.01**	**2.6 (1.4-5.0)**	10 (8.1%)	**0.003**	**3.6 (1.7-8.0)**	4 (2.5%)	1.00	0.9 (0.3-2.7)
G/G-C/C	6 (0.7%)	4 (0.9%)	0.74	1.3 (0.4-4.6)	2 (0.7%)	1.00	1.1 (0.2-5.3)	2 (1.6%)	0.24	2.6 (0.5-12.9)	2 (1.2%)	0.63	1.6 (0.3-8.0)
G/G-C/T	2 (0.2%)	1 (0.2%)	1.00	1.0 (0.1-10.6)	0 (0.0%)	-	-	0 (0.0%)	-	-	1 (0.6%)	0.44	2.4 (0.22-26)
G/G-T/T	0 (0.0%)	1 (0.2%)	-	-	1 (0.4%)	-	-	1 (0.8%)	-	-	0 (0.0%)	-	-

*BC* breast cancer, *OC* ovarian cancer, *OR* odds ratio, *CI* confidence interval.

^a^Fisher’s exact test.

Bold values are statistically significant (*P* < 0.05).

Although phase for these variants could not be determined directly from the screening data, haplotypes were constructed from genotype data using UNPHASED software, which uses a maximum-likelihood approach. Theoretically, the total number of haplotypes with two SNPs is four, but we observed only three: wildtype (rs152451-A - rs45551636-C), rs152451-G - rs45551636-C, and rs152451-G - rs45551636-T. The haplotype estimation suggested a strong linkage disequilibrium between the two markers (coefficient of linkage disequilibrium, D' = 1). When we analyzed the haplotypes and BC risk considering the number of BC cases in the families (Table 5), we observed that rs152451-G - rs45551636-C and rs152451-G - rs45551636-T haplotypes were associated with a significantly increased BC risk in the families with a strong family history of BC and/or OC (OR = 1.6 [CI 95% 1.0-2.5] p = 0.05 and OR = 3.7 [CI 95% 1.8-7.5] p < 0.001, respectively). No association was observed in the families belonging to subgroup B.

**Table 5:** **Haplotype effects for rs152451 and rs45551636** ***PALB2*** **variants**

		All BC cases (n = 436)			Families with ≥ 2 BC and/or OC cases (n = 273)			Families with ≥ 3 BC and/or OC cases (n = 124)			Single affected, diagnosis ≤ 50 years (n = 163)		
Haplotype	Controls (%) (n = 809)	BC cases (%)	*P*value^a^	OR [95% CI]	BC cases (%)	*P*value^a^	OR [95% CI]	BC cases (%)	*P*value^a^	OR [95% CI]	BC cases (%)	*P*value^a^	OR [95% CI]
A-C	1475 (91.2%)	781 (89.6%)	-	1.0 (1.0-1.0)	480 (87.9%)	-	1.0 (1.0-1.0)	209 (84.3%)	-	1.0 (ref)	301 (92.3%)	-	1.0 (1.0-1.0)
G-C	120 (7.4%)	67 (7.7%)	0.75	1.1 (0.8-1.4)	47 (8.6%)	0.31	1.2 (0.8-1.7)	27 (10.9%)	**0.05**	**1.6 (1.0-2.5)**	20 (6.1%)	0.48	0.8 (0.5-1.3)
G-T	23 (1.4%)	24 (2.8%)	**0.03**	**2.0 (1.1-3.5)**	19 (3.5%)	**0.004**	**2.5 (1.4-4.7)**	12 (4.8%)	**<0.001**	**3.7 (1.8-7.5)**	5 (1.5%)	0.80	1.1 (0.4-2.8)

*BC* breast cancer, *OC* ovarian cancer, *OR* odds ratio, *CI* confidence interval.

^a^Fisher’s exact test.

Bold values are statistically significant (*P* < 0.05).

### Prediction of functional effect of PALB2 variants

The variant c.1861C > A produces the change of Pro to Met at position 621. The multiple alignment analysis with the *PALB2* protein sequence indicates that the amino acid in position 621 is conserved in various species. The case-control study showed that this variant was detected in only one of 436 cases and none of the 809 controls. This results suggest that the variant c.1861C > A could be pathogenic. In order to predict the possible effect of this amino acid change in the structure and function of the *PALB2* protein, we used PolyPhen-2, SIFT and PROVEAN software, which predicted that this variant is not likely to be pathogenic. To evaluate protein stability, we used the i-Mutant program. The amino acid change decreases the protein stability (-0.55 Kcal/mol). Considering the case-control study and *in silico* analyses, the c.1861C > A missense mutation can be classified as a variant of unknown significance (VUS), or as a rare variant.

The variant c.1676A > G (p.Q559R) corresponds to a previously-described variant (rs152415). The three pathogenicity programs predicted that it is likely non-pathogenic. The i-Mutant program shows that the amino acid change slightly decreases the protein stability (-0.06 Kcal/mol). The variant c.2993C > T (p.G998E) is also a previously-described variant (rs45551636). The results of the bioinformatic predictor programs indicated that it is probably pathogenic. The results obtained with the i-Mutant program predicted that the mutation decreases the protein stability (-0.41 Kcal/mol). In order to analyze the possible effect of this variant on the 3D-structure and function of the *PALB2* protein, we carried out homology modeling using MODELLER v.9.12 (Additional file 2). The model obtained was compared in RASMOL with the structure of the wildtype protein, with results predicting that the amino acid change might affect this region and then modify *PALB2* function. Considering there are currently no published functional studies, this variant could be classified as a variant of unknown significance.

## Discussion

Mutations in *BRCA1* and *BRCA2* are associated with susceptibility to breast and ovarian cancer. At present, however, those mutations account for only a portion of familial cases, and consequently there is an intensive search for additional targets. *PALB2* mutations have been identified as BC susceptibility alleles by both case-control and family studies [9,10,13,17,21,28-35], and are closely associated with *BRCA2*. Since the initial identification of *PALB2* as a BC susceptibility gene [9], several investigators have screened for the gene in *BRCA1/2*-negative families with BC and/or women diagnosed with early-onset BC from various ethnic backgrounds, such as Australian [12,36], Chinese [16], German [32], Italian [33,37,38], Dutch [39], North American [17,21,40-44], Polish [31], Russian [32], South African [45], and Spanish [46,47] populations. However, the prevalence of *PALB2* variants in the South American population had not yet been established. In the present study, we analyzed the complete coding sequence and exon-intron boundaries of *PALB2* in Chilean *BRCA1/2*-negative BC patients; nevertheless, no pathogenic mutations were observed in the *PALB2* coding sequence. Pathogenic mutations in *PALB2* are rare (varying from 0.1% to 2.7%) and vary in frequency depending on the population [48]. No *PALB2* germline mutations have been observed in the geographically-confined population of Iceland, for instance [49]. The contemporary Chilean population stems from the admixture of Amerindian peoples with the Spanish settlers in the 16th and 17th centuries. Later migrations in the 19th century of other populations (e.g., Germans, Italians, Arabs, and Croatians) have had only a minor impact on the overall population (≤4% of the total population) and are restricted to the specific locations of the country where they originally settled [50]. The relationships among ethnicity, Amerindian admixture, genetic markers, and socioeconomic strata have been extensively studied in Chile [51-53]. Thus, it is probable that in the mixed Chilean population, *PALB2* is not a significant contributor to BC in high-risk BC families.

We identified three sequence variants: c.1676A > G (exon 4), c.1861C > A (exon 5), and c.2993C > T (exon 9). With respect to c.1861C > A (exon 5), this variant is not previously described in the literature or in the *PALB2* mutation and polymorphism database (http://www.lovd.nl/PALB2). It corresponds to a missense mutation that produces the change of proline to methionine in the 621 position (p.P621M). It was found in only one of the 436 cases and in none of 809 controls. The patient carrier of this variant belongs to a *BRCA1/2*-negative family with three BC cases. Three sisters were screened for the variant, two with BC (diagnosed at 45 and 49 years) and the other healthy. The c.1861C > A carrier was the sister with BC with the earliest age of diagnosis. The bioinformatic analyses using PolyPhen-2, SIFT and PROVEAN tools predicted that this variant is not pathogenic, and therefore we classified this missense mutation as a new mutation of unknown significance.

The variants c.1676A > G (p.Q559R) and c.2993C > T (p.G998E) correspond to previously-described variants (rs152451 and rs45551636, respectively). These have been described in Spanish [46], Australian [29], Asian (Malaysian and Singaporean) [15], African–American [54], German and Russian [32], Italian [33], Chinese [16], and Finnish [10] populations. It is widely accepted that according to bioinformatic analyses, the c.1676A > G variant corresponds to a benign polymorphism while c.2993C > T affects *PALB2* protein function. In the present study, using a case-control design, we evaluated the impact of *PALB2* rs152451 and rs45551636 variants in Chilean women with familial and non-familial early-onset BC who are negative for *BRCA1/2* point mutations. In both cases and controls, the allelic frequencies of both variants were similar to those reported by Blanco et al. (2013) [46] in a Spanish population. The MAF of rs152451 and rs45551636 in cases (0.10 and 0.03, respectively) differed from those reported in German (0.06 and 0.03, respectively) [32], Russian (0.06 and 0.01, respectively) [32], and African-American (0.16 and 0.005, respectively) [54] populations. In controls, the MAF of rs152451 and rs45551636 were 0.09 and 0.01 respectively, differing from those reported in the NHBLI ESP6500 database for African-Americans (0.22 and 0.006 respectively) but similar to those reported for European-Americans (0.09 and 0.02 respectively). Therefore, MAF frequencies vary by ethnicity. In the present study, both variants were significantly associated with increased risk of familial BC but not non-familial early-onset BC. On the other hand, when we consider the number of BC cases in the family, we found a significantly increased BC risk only in the carriers of allele G (AG + GG) in families with 3 or more members affected with BC and/or OC (OR = 2.0 [CI 95% 1.3-3.2], p < 0.01). Therefore, we propose that these two variants are associated with increased BC risk in families with a strong family history of BC. These results are in agreement with those published by Antoniou et al. [55], who concluded that the pathogenic mutations in *PALB2* confer an absolute risk of 58% for those women with two or more first-degree relatives with BC at 50 years of age, and that the risk is lower for women with no family history of BC. This result was confirmed by the composite genotype analysis, suggesting that these two variants could be in linkage disequilibrium. The haplotype analysis also suggested strong linkage disequilibrium between the two markers. Also, we observed that rs152451-G/rs45551636-C and rs152451-G/rs45551636-T were associated with significantly increased BC risk in families with a strong family history of BC. In the literature, there are no publications with the analysis performed in the present manuscript.

The prediction of functional effects revealed that rs152451 is probably non-pathogenic. Nevertheless, the region of this variant is found inside the *PALB2* motif, which interacts with chromatin [56]. This region was not crystallized; therefore, no 3D-structure information is available to evaluate the possible impact of this mutation. However, the analyses of the amino acid change properties (p.Q559R) lead us to propose that this variant might have an impact on *PALB2* function. Functional analyses are needed to elucidate the effect of c.1676A > C on *PALB2* protein function, and thus to confirm the results obtained in the case-control study. Regarding rs45551636, the bioinformatic analysis using PolyPhen-2, SIFT and PROVEAN tools predict that this variant is probably pathogenic. c.2993C > T is located within the *PALB2* WD40-domain, which is involved in the interaction of the *PALB2* protein with *BRCA2* and *RAD51* [56]. To analyze the effect of the amino acid change on *PALB2* protein function, the model obtained using MODELLER v.9.12 was compared with the wildtype *PALB2* structure. We observed that the wildtype structure Gly, which is smaller and very flexible, takes part in the loop between WD 2 and 3 repetitions. On the other hand, in the mutated structure, glutamic acid, which is more voluminous and negatively-charged, is protruded towards the interior of the hydrophobic pocket formed by WD40 repetitions resulting in an unusual torsion angle localized at the loop that connects these regions. In addition, with the evidence obtained from the bioinformatic tools, the structure-modeling analysis strongly suggests that this variant could affect the protein function.

## Conclusions

Our data shows that *PALB2* pathogenic germline mutations are not present in a Chilean population, and that rs152451 and rs45551636 are associated with increased BC risk in *BRCA1/2*- negative families with a strong family history of BC but not in non-familial early-onset BC. These results are in agreement with Southey et al. (2010) [12].

## Additional files

Additional file 1: Table S1. Primer sequences to amplify the whole coding sequence and exon–intron boundaries of the *PALB2* gene.
Additional file 2:
**Comparison of the WD40 domain’s three-dimensional structure in the wildtype PALB2 protein and the p.G998E (c.2993C > T) variant.**
