# Supplementary material for: Association of PALB2 sequence variants with the risk of familial and early-onset breast cancer in a South-American population
Source: BMC Cancer. 2015 Jan 31;15:30. doi: 10.1186/s12885-015-1033-3 (PMC4323211; doi:10.1186/s12885-015-1033-3)
Supplement: Additional file 1: Table S1. — Primer sequences to amplify the whole coding sequence and exon–intron boundaries of the PALB2 gene. [file 12885_2015_1033_MOESM1_ESM.doc]

Supplementary Table S1. Primer sequences to amplify the whole coding sequence and exon–intron boundaries of the *PALB2* gene.

| Exon | Sequence | Amplicon size, bp |
| --- | --- | --- |
| 1 | F: GTGCGCAGGCCGAATGGT | 286 |
|  | R: AGTCCTGCGTCCGCCCTTC |  |
| 2,3 | F: GTGCTACTCCCTGCCTCTTG | 471 |
|  | R: CACACTGTGGGAAAAAGAACAA |  |
| 4a | F: TGTCACTGATTCTTTCTTAAATAAATGTT | 594 |
|  | R: TGGGCAGTTGGTGGAATTA |  |
| 4b | F: GCAAAAATCCTGCTAGATCACC | 556 |
|  | R: AGATTTTCATTCCTGCCATCA |  |
| 4c | F: CCAGCAAATGAAAACCAAAA | 396 |
|  | R: GCATGTGCCAGACATCCTAA |  |
| 4d | F: GAGTCATTTGGATGTCAAGAAAAA | 470 |
|  | R: AAGGAAGTGCCAGGCAAATA |  |
| 5a | F: TTGTCTGTTTTGTTGGGTTTTG | 507 |
|  | R: GCGCAACCGTATTTAAAGGA |  |
| 5b | F: ACATCCCAAAAGGCCAAACT | 569 |
|  | R: TAAACGTGGAAGGCCCAAT |  |
| 6 | F: ATTTGGAGCTTTGCTGCTGT | 275 |
|  | R: TGACTGAATTCTTTTCAGTTCATT |  |
| 7 | F: TGCTTTGCATAAAACAGCACT | 315 |
|  | R: GCATGGTCATAGCTCCCAAT |  |
| 8 | F: CAAAAATGAAACAACCAAGCA | 203 |
|  | R: TGCACTTAAAACCAGCTGACA |  |
| 9 | F: ACCCCGTCTCTACAAAAATAC | 540 |
|  | R: ATTACACCCCCAGCACAGAA |  |
| 10 | F: CAGTTCAACAATGCGGAGAA | 278 |
|  | R: TCTTCACAACAACCCTGTAAAA |  |
| 11 | F: TTTCCCTGGTCACCTCCTAA | 290 |
|  | R: CGGGGAAGGTTTGTTCATTA |  |
| 12 | F: TTGTTTGGTTTTTGTCTCTGC | 259 |
|  | R: TGTGTTTGCACAGTGCCTTT |  |
| 13 | F: TGGGAACATGGTTTTGACCT | 643 |
|  | R: TGCTTCTGCAAATGATCTTGA |  |
