# Supplementary material for: Association of PALB2 sequence variants with the risk of familial and early-onset breast cancer in a South-American population
Source: BMC Cancer. 2015 Jan 31;15:30. doi: 10.1186/s12885-015-1033-3 (PMC4323211; doi:10.1186/s12885-015-1033-3)
Supplement: Additional file 2: — Comparison of the WD40 domain’s three-dimensional structure in the wildtype PALB2 protein and the p.G998E (c.2993C > T) variant. [file 12885_2015_1033_MOESM2_ESM.pdf]

Additional file 1

Comparison of the WD40 domain's three-dimensional structure in the wildtype PALB2 protein and the p.G998E (c.2993C>T) variant

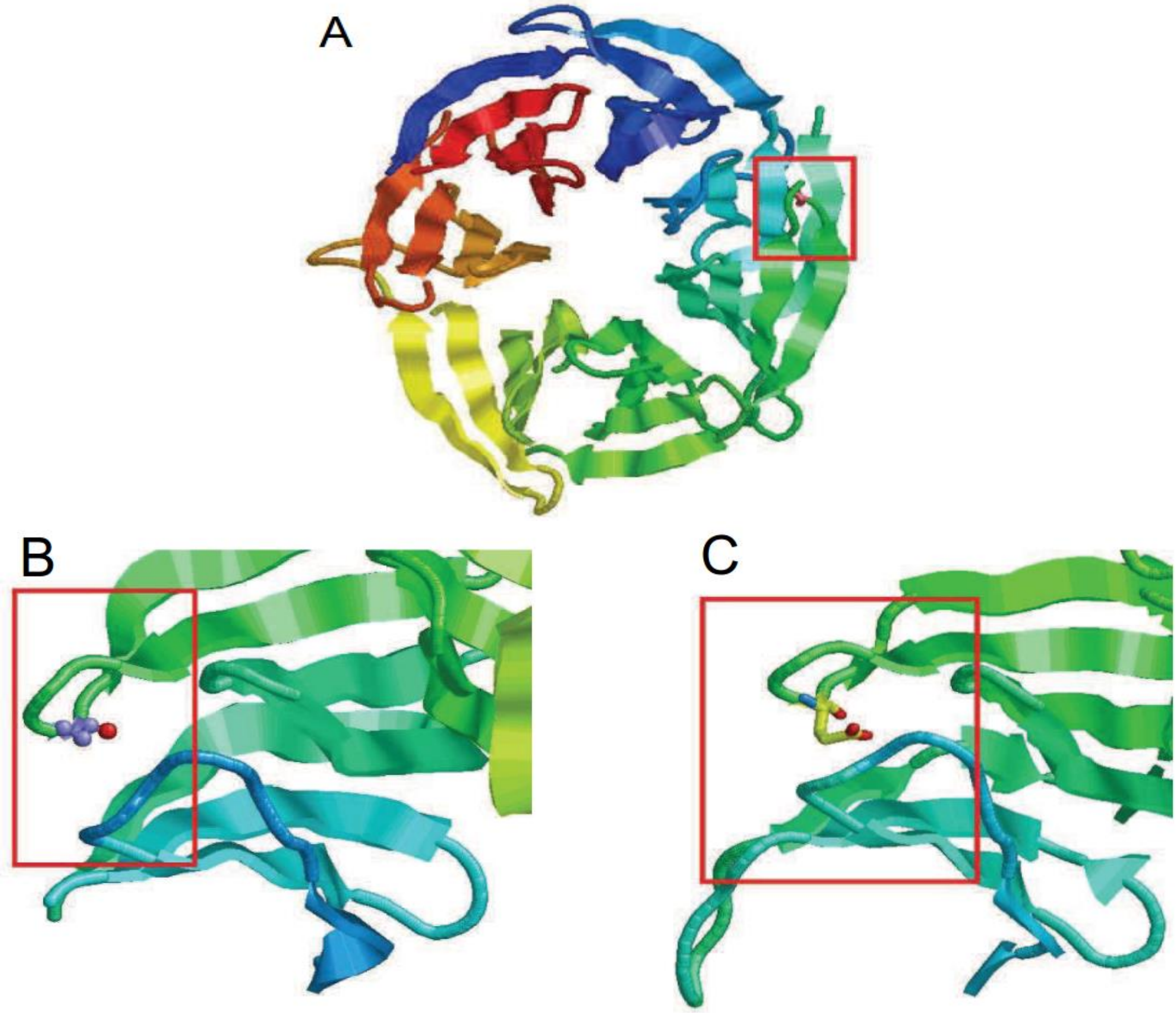

A. Three-dimensional structure of the WD40 domain of PALB2. The red box indicates the position of the rs4551636 (p.G998E) variant between repetitions of WD 2 and 3, colored turquoise and green, respectively. B. Magnification of the location of the 998 glycine amino acid in the wildtype protein. C. Amplification of the region where the 998 amino acid is located in the structure predicted for the protein with the amino acid variation.
